# Expression of toll-like receptors and inflammatory mediators in primary human glioma cells (low- and high-grade) compared to primary cells from epileptic human brain

**DOI:** 10.22038/ijbms.2025.83939.18166

**Published:** 2025

**Authors:** Negin Masoomabadi, Safieh Ebrahimi, Farshid Noorbakhsh, Alireza Tabibkhooei, Gisou Mohaddes, Saeed Sadigh-Eteghad, Maryam Khaleghi Ghadiri, Tahereh Ghadiri, Ali Gorji

**Affiliations:** 1 Department of Neuroscience and Cognition, Faculty of Advanced Medical Sciences, Tabriz University of Medical Sciences, Tabriz, Iran; 2 Department of Clinical Biochemistry, Faculty of Medicine, Mashhad University of Medical Sciences, Mashhad, Iran; 3 Shefa Neuroscience Research Center, Khatam Alanbia Hospital, Tehran, Iran; 4 Skull Base Research Center, Department of Neurosurgery, Iran University of Medical Sciences, Tehran, Iran; 5 Neuroscience Research Center, Tabriz University of Medical Sciences, Tabriz, Iran; 6 Department of Biomedical Education, College of Osteopathic Medicine, California Health Sciences University, Clovis, CA, USA; 7 Epilepsy Research Center, Neurosurgery Department, Münster University, Münster, Germany; 8 Neuroscience Research Center, Mashhad University of Medical Sciences, Mashhad, Iran

**Keywords:** Glioma, High-grade glioma Inflammation, Low-grade glioma, Toll-like receptors

## Abstract

**Objective(s):**

Toll-like receptors (TLRs) have been implicated in the pathogenesis of glioma as principal regulators of inflammation and innate immune function. Considering the heterogeneous nature of gliomas, ranging from low to high grade with different therapeutic responses, investigating the differences in the levels of TLR2 and TLR4 and associated inflammatory markers in these distinct groups is of great clinical significance.

**Materials and Methods:**

In this study, we investigated changes in the protein expression levels of TLR2 and TLR4, along with key inflammatory mediators, including nuclear factor kappa B (NF-κB) as a downstream signaling molecule, and tumor necrosis factor-alpha (TNF-α), a target of NF-κB activation, by using western blotting. Primary human cells were isolated from surgically resected tissue samples of patients with low- and high-grade gliomas and were compared to cells derived from the human epileptic brain. *In vitro* cell characterization was performed via immunocytochemistry using markers specific to each group.

**Results:**

Protein levels were assessed by western blotting. TLR2 expression was significantly higher in the high-grade glioma group compared to the low-grade group. The expression of TLR2 and TLR4 was significantly greater in the epilepsy group compared to the low-grade glioma group. No remarkable differences were detected in the levels of NF-κB and TNF-α between high and low-grade gliomas.

**Conclusion:**

Our results revealed distinct patterns of TLR expression between low- and high-grade gliomas, underscoring the potential involvement of TLRs in the cellular heterogeneity of gliomas.

## Introduction

Gliomas represent the most prevalent primary tumors of the central nervous system (CNS) with high mortality rates and a heterogeneous nature from low to high grade (1-3). High-grade gliomas (HGGs) are categorized as World Health Organization (WHO) grades III and IV, while low-grade gliomas (LGGs) fall under WHO grades I and II (2). HGGs are the most aggressive form of brain tumor, marked by rapid disease progression, frequent recurrence, and a poor survival prognosis (4). The 2-year survival rate is below 5%, while the median overall survival duration is 14.6 months (5). Compared with HGG patients, LGG patients have a relatively better prognosis with a median overall survival of 1 to 15 years (6). However, most of these patients are prone to transformation to HGG (7). Consequently, understanding the molecular mechanisms underlying glioma pathogenesis, particularly the transition from LGG to HGG, is crucial. Neuroinflammation is recognized as a key factor in glioma tumorigenesis and progression (7-9). The tumor microenvironment in gliomas exhibits complex immune interactions, where Toll-like receptors (TLRs) are pivotal regulators of both pro-tumoral and anti-tumoral responses (10-12). TLRs are members of pattern recognition receptors acting as the primary line of defense in the innate immune system diagnosing the pathogen-associated molecular patterns (PAMPs), like lipopolysaccharide (LPS)(13, 14). 13 different TLRs (TLR1-13) are expressed in humans, among which TLR2 and TLR4 have been extensively studied for their involvement in inflammation and cancer development (15). TLR2 and TLR4 are overexpressed in many tumor types and act as proto-oncogenes by affecting tumor cell proliferation, migration, and metastasis (16). Additionally, TLR4 has been reported to play a key role in nuclear factor kappa B (NF-κB) activation and subsequent production of pro-inflammatory cytokines, including interleukins and tumor necrosis factor-α (TNF-α)(17). The overexpression of TLR2 and TLR4 has been previously documented in glioma cell lines and tissue samples, with both receptors implicated in promoting glioma tumorigenesis (18-20). Increased expression of TLR9 has also been associated with higher tumor grade (21). Despite the established role of TLR in gliomas, three key mechanistic gaps remain unaddressed: (i) The differential expression patterns of TLR2/4 across glioma grades remain poorly characterized; (ii) The functional consequences of TLR signaling in grade-specific contexts are unclear; and (iii) The potential of TLR expression profiles to distinguish tumor-associated neuroinflammation from non-tumor conditions, such as epilepsy, needs further investigation. Our study addresses these shortcomings through a comparative analysis of primary human cells derived from human LGG, HGG, and epileptic brain tissues. 

## Materials and Methods

All investigations were carried out following the National Institute of Health Guidelines for the Care and Use of Laboratory Animals and received approval from the Ethics Committee of the Shefa Neuroscience Research Center in Tehran, Iran. Informed consent was secured from all participants.

### Primary cell culture

The primary human cells were obtained from fresh surgically resected tissue samples of 5 patients with epilepsy, five patients with low-grade glioma, and 5 cases with high-grade glioma. Table 1 provides a summary of the patient’s information. Samples of the patients were washed in Phosphate-buffered saline (PBS) until all the blood contaminants were removed. Tissue specimens were then processed by mechanical homogenization using a sterile scalpel and transferred to a 50 ml Falcon tube containing 0.05% Trypsin/EDTA (DNAbiotech Co., Tehran, Iran) and maintained at a 37 ^°^C water bath for about 15 min. Afterward, the cell suspension was centrifuged at 1200 rpm for five minutes, and the cell pellets were resuspended in DMEM/F12 medium (DNAbiotech Co., Tehran, Iran) supplemented with FBS fetal bovine serum (FBS)(Gibco). For epilepsy and low high-grade glioma samples, a complete medium composed of (DMEM/F12, supplied with 10% FBS (Gibco), 1% penicillin-streptomycin (DNAbiotech), 1% L-glutamine (Gibco)) was employed. Subsequently, the cell suspension was titrated via pipetting and filtered with a 70 μm cell strainer. Finally, the obtained cells were resuspended in the related proliferation medium and cultured as a monolayer in a T25 cell culture flask. All cultures were kept at 37 ^°^C in a humidified incubator with 5% CO_2_ and were sub-cultured when they reached 80% confluency.

### Immunocytochemical analysis

*In vitro* characterization of primary human glioma and epileptic cells was assessed by immunohistochemistry using markers specific for each cell. Briefly, Cells were fixed in 4% paraformaldehyde in PBS for 15 min at room temperature and then washed three times with PBS for 10 min. Cells were then incubated for 30 min with a blocking buffer containing 10% NGS and 0.1% Triton X-100 in PBS. Cells were incubated overnight with primary antibodies, including Nestin (1:200, rabbit polyclonal, Sigma) and Ki-67 (1:100, rabbit polyclonal, Abcam), diluted in PBS with 1% BSA, 5% FBS, and 0.1% Triton X-100. The following morning, the cells underwent three washes with PBS. They were then incubated with secondary antibodies (1:200, FITC-conjugated polyclonal goat anti-rabbit antibody) diluted in PBS with BSA 1%, and 0.1% Triton X-100 for one hour at room temperature with gentle agitation. Ultimately, cells were counterstained with DAPI (1:1000) for five minutes and washed with PBS. Pictures were obtained by a fluorescent microscope.

### Western blot analysis

Cell lysates were obtained by carefully scraping the cells in a lysis buffer composed of 1% Triton, 0.1% SDS, 50 mM TRIS-HCl, 150 mM NaCl at pH 7.4, and 2 mM EDTA, along with a protease inhibitor. The resulting lysates underwent centrifugation at 12,000 rpm for 15 min at 4 ^°^C. Protein concentrations in the lysates were measured using the Bio-Rad Protein Concentration Assay. Following this, 100 μg of the lysate samples were resolved by molecular weight on 10% SDS-PAGE gels and transferred to a nitrocellulose membrane. The membranes were blocked with 5% non-fat dry milk at room temperature for one hour and then incubated overnight at 4 ^°^C with primary antibodies diluted in 5% BSA and 0.1% Tween 20 in TRIS-buffered saline. The primary antibodies used included rabbit anti-NF-κB P65: orb312399 (1:500 diluted) (Biorbyt), rabbit anti-TNFα: orb214681 (1:500 diluted) (Biorbyt), rabbit anti-TLR2: orb11487 (1:500 diluted) (Biorbyt), rabbit anti-TLR4: orb11489 (1:500 diluted) (Biorbyt), mouse anti-ß-actin sc-47778 (1:500 diluted), and rabbit anti-GAPDH antibody GTX100118 (1:5000 diluted). A secondary antibody (rabbit): BA1054-2 was used for detection. Protein bands were revealed using an enhanced chemiluminescence (ECL) detection system or DAPI, and quantification was done through densitometry using Image J software. 

### Statistical analysis

All experiments were conducted in triplicate and analyzed with GraphPad Prism v8 software. Results are depicted as mean±standard deviation (SD). A one-way ANOVA was applied for group comparisons, with a *P*-value less than 0.05 considered statistically significant.

## Results

### In vitro cell characterization

**Figure 1:**
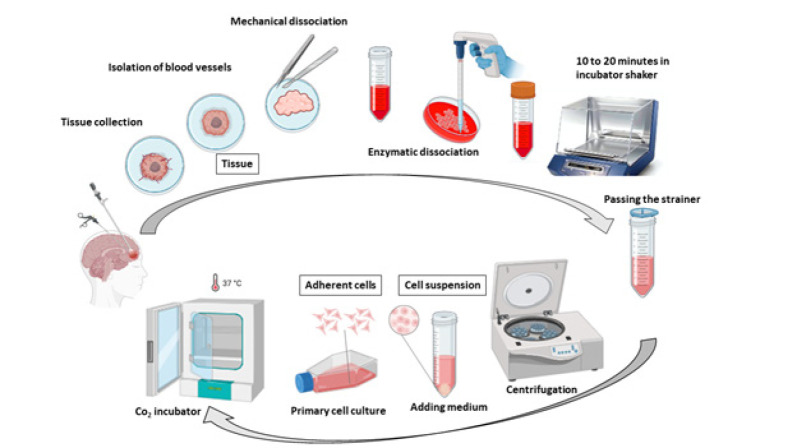
**. **A schematic representation of the establishment of primary human cells obtained from fresh surgically resected tissue

**Figure 2:**
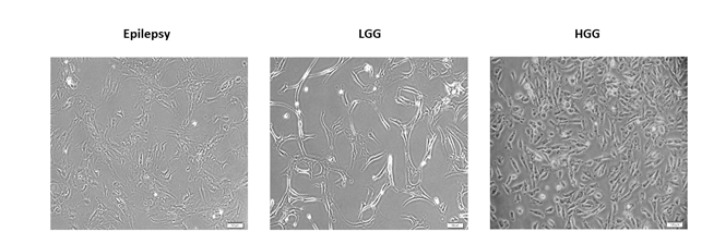
A representative morphological picture of the primary human cells isolated from fresh surgically resected tissue samples of patients with epilepsy, low-grade glioma (LGGs), and high-grade glioma (HGG)

**Figure 3:**
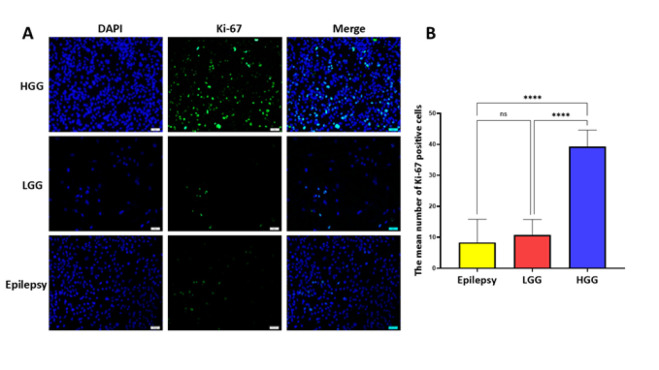
A) Immunocytochemistry analysis of ki-67 (green) expression in primary human cells of patients with epilepsy, low-grade glioma (LGGs), and high-grade glioma (HGG). Nuclei were stained with DAPI (blue). The merged images are also shown in the final column: scale bar, 50 μm. B) The mean number of ki-67 positive cells was measured by ImageJ software, plotted, and compared. ** *P≤*0.01; *** *P≤*0.001, and **** *P≤*0.0001

**Figure 4:**
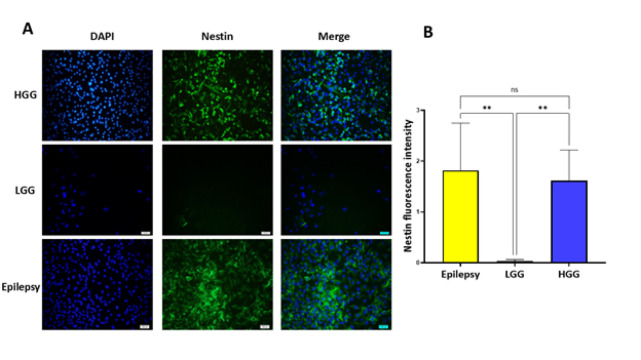
A) Immunocytochemistry analysis of Nestin (green) expression in primary human cells of patients with epilepsy, low-grade glioma (LGG), and high-grade glioma (HGG). Nuclei were stained with DAPI (blue). The merged images are also shown in the final column: scale bar, 100 μm. B) The Nestin fluorescence intensity was evaluated using ImageJ software, and the results were plotted and compared

The primary human cells were obtained from fresh surgically resected tissue using a procedure based on tissue mechanical disaggregation followed by enzymatic digestion, cell centrifugation, and purification. A schematic representation of the establishment of primary human cells is shown in Figure 1. A representative morphological picture of the primary human cells of patients with epilepsy, HGG, and LGG is also shown in Figure 2. To further characterize the primary human glioma and epileptic cells, the expression of specific marker proteins was assessed via immunocytochemistry. Protein markers, including Ki-67 and Nestin, were used for *in vitro* characterization. Ki-67 is a cell proliferation marker highly associated with glioma tumorigenesis and histologic grade. Ki-67 is being extensively employed to determine tumor cells and the histological grade of the tumor, as Ki-67 expression is positively correlated with tumor aggressiveness and grade (22). We observed higher Ki-67 positive cells in the HGG group compared to the LGG and epilepsy groups, confirming these cells’ nature (Figure 3). Nestin is an intermediate filament component in embryonic development, expressed temporarily by precursor cells that will differentiate into neurons and glia in the brain. Nestin is a specific marker of mature astrocytes and is expressed in reactive astrocytes in some pathological conditions, including epilepsy (23). As shown in Figure 4, the majority of cells in epilepsy groups are Nestin-positive, while a few Ki-67-positive cells were observed. Furthermore, several studies have found that Nestin expression is related to the degree of glioma malignancy (24). We consistently found higher Nestin-positive cells in the HGG group compared to the LGG group. Collectively, both the Ki-67 and Nestin data confirm the nature of these cells.

### Expression of inflammatory mediators

**Figure 5:**
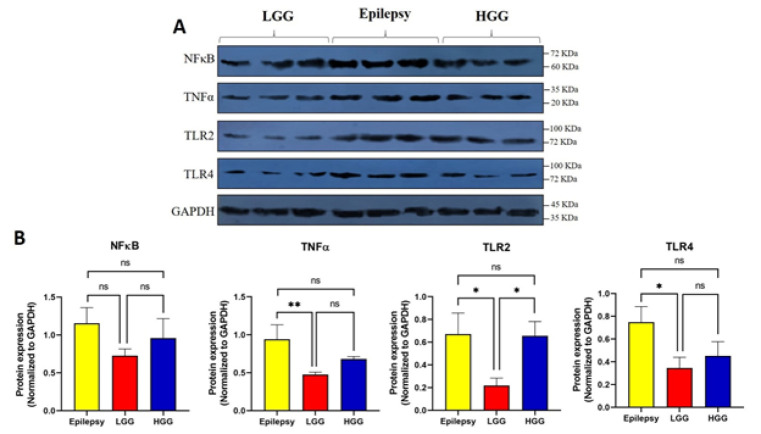
Protein expression of inflammatory mediators in primary human cells derived from low-grade glioma (LGG), high-grade glioma (HGG), and epilepsy tissues

We performed Western blot analysis to measure TLR2, TLR4, NF-κB, and TNF-α protein levels in primary human cells derived from fresh surgically resected tissue samples of patients with HGG, LGG, and epilepsy. As illustrated in Figure 5, TLR2 expression was significantly higher in both HGG and epilepsy tissues than in LGG, with no significant difference between HGG and epilepsy (*P*<0.05). For TLR4, there was a trend toward higher expression in HGG compared to LGG, although this difference did not reach statistical significance. However, TLR4 expression was significantly higher in epilepsy tissues compared to LGG (*P*<0.05), and not significantly different between epilepsy and HGG (Figures 5A and B).

TNF-α expression was also significantly increased in human epileptic brain samples compared to LGG tissues (*P*<0.01), with no significant differences between HGGs and LGGs. NF-κB levels did not show statistically significant differences among the three groups, although a trend toward increased expression in epileptic tissues and HGGs was observed (Figures 5A and B).

## Discussion

In the present research, we have specifically explored the differences in the levels of TLR2 and TLR4 and associated inflammatory markers in primary human cells derived from fresh surgically resected tissue samples of patients with LGGs and HGGs, as well as patients with epilepsy as a nontumor intracranial disease. Our research presents three key findings: (i) TLR2 expression increases significantly with tumor grade, with HGGs showing greater levels than LGGs; (ii) epileptic tissues exhibit TLR2/4 overexpression compared to LGGs; and (iii) NF-κB and TNF-α expression does not align with that of the TLRs in tumor tissues, suggesting pathway-specific activation patterns.

Growing evidence highlights a significant relationship between inflammation and tumor grade, suggesting that inflammatory processes may play a key role in tumor progression and malignancy (25). Yet few studies have compared specific inflammatory markers between LGGs and HGGs (8, 9, 26-28). Measuring inflammatory marker levels may aid in assessing disease progression and predicting glioma grade, highlighting their potential as biomarkers for identifying more aggressive tumor types (8, 9). TLRs regulate host defense mechanisms, elicit pro-tumorigenic responses, and could be deemed potential biomarkers to monitor tumor progression, specifically TLR4 in tumor survival and chemoresistance, and TLR2 in metastatic progression (29-32). Given that TLRs are closely related to tumor development and grade, we explored whether there is any difference in TLR2 and TLR4 expression patterns between LGGs and HGGs. The expressions of TLR2 and TLR4 have been previously examined in clinical glioma tissues and glioblastoma U87 cell lines (18-20). TLR2 and TLR4 were shown to be up-regulated in glioma samples and are negatively correlated with poor prognosis (20, 33). In this study, we used human primary cells isolated directly from tissues instead of cell lines, enhancing the physiological relevance of the *in vitro* and *in vivo* data obtained (34). While cell lines provide a rapid and reliable approach for *in vitro* investigations, they do not accurately represent the heterogeneous characteristics of tumors *in vivo* (35). Studies have demonstrated that glioma cell lines display unique gene expression profiles in contrast to primary tumors, and are susceptible to genetic alterations over successive passages, which can affect the reliability of experimental results (36). However, primary cells maintain molecular and cellular characteristics identical to the original tumor mass, which could be a promising source for investigation. We found that primary cells derived from patients with HGGs exhibited significantly higher levels of TLR2 expression compared to LGGs. These findings suggest the potential of using TLR2 expression as a marker for monitoring disease progression and investigating inflammatory mechanisms in glioma. Consistent with our results, Li and colleagues demonstrated a significant increase in TLR2 expression in HGGs compared to LGGs, noting a positive correlation between TLR2 expression and tumor grade, and a role in promoting glioma development through enhanced autophagy (33).

Furthermore, we observed a trend toward increased TLR4 expression in HGG tissues compared to LGGs, although this difference did not achieve statistical significance. This upward trend may still be biologically relevant, and the absence of statistical significance could be attributed to the relatively small sample size, which may have limited the power to identify subtle variations. Notably, TLR4 expression was significantly higher in epileptic tissues than in LGG, suggesting that elevated TLR4 may be associated with inflammatory conditions beyond tumor grade alone. This observation is consistent with existing data indicating that TLR4 expression is context-dependent and influenced by tumor microenvironment, immune infiltration, and glioma subtype. TLR4 expression in gliomas has been variably reported in the literature. Some studies have shown downregulation of TLR4 in human U87MG glioma cell lines and human specimens resected from 8 patients with primary brain tumors (37). TLR4 downregulation is suggested as a mechanism by which tumors escape from the anti-tumor immune responses. TLRs have a dual regulatory function in tumors, exhibiting both anti-tumoral and pro-tumoral effects (38). TLRs promote anti-tumoral effects through the direct induction of tumor cell apoptosis and the stimulation of anti-cancer immune responses. Therefore, down-regulation of TLR4 leads to immune system evasion. TLR4 expression is cell type and stage-specific and can be affected by the different tumorigenic and metastasis processes, immune capacities of cancer cells, and particularly differences in the immune cells infiltrating the tumor (38). For instance, glioma grade IV has been shown to express lower levels of TLR4 compared to grade II-III gliomas. This finding was associated with increased hematopoietic-derived immune cells (37). Therefore, further studies are required to unravel the complexities of TLR expression patterns and TLR-regulating factors for improving potential TLR-targeted anti-tumor therapies. 

In addition to glioma tissues, we observed significantly higher expression of TLR2 and TLR4 in epilepsy-derived tissues than in LGGs. This suggests TLRs may help differentiate neoplastic from non-neoplastic intracranial conditions (39). It has been evidenced that in instances of cellular damage and epileptic seizures, TLR4 is prominently expressed in neurons, astrocytes, and microglia. Studies have shown that TLR4 activation is linked to the onset of seizures. At the same time, its inhibition or removal leads to a decrease in both acute and chronic seizure occurrences in mouse models of epilepsy (40, 41). Pre-clinical investigations have revealed that elevated levels of TLR4 correlate with a heightened risk and severity of epilepsy, along with a greater probability of developing resistance to anticonvulsant treatments (42). Kamaşsk and colleagues found that TLR4 levels were remarkably greater in patients with severe epilepsy, suggesting a relationship between epilepsy severity and TLR4 expression levels (43). In another pre-clinical study utilizing a mouse model of mesial temporal lobe epilepsy, overexpression of the HMGB1/TLR4 axis was noted, which has the potential to induce synaptic remodeling and inflammatory responses in neurons via the p38MAPK signaling pathway (44). TAK-242, a selective TLR4 inhibitor, has been shown to attenuate epileptic symptoms, presumably through suppressing the TLR4/NF-κB signaling axis (45). Pernhorst et al. found that elevated TLR4 gene expression is associated with an increased frequency of seizures (46). These findings support the role of TLR4 in epileptogenesis and inflammation-driven neuronal remodeling. 

NF-κB activation is closely linked to the malignant properties of tumor cells during glioma progression and is an important downstream mediator of TLR-induced tumorigenesis (47). This pathway has been shown to be activated and correlated with higher tumor grades across various types (42). Considering the downstream signaling of TLRs, we further analyzed NF-κB and TNF-α expression levels. We found no significant alterations in the levels of NF-κB and TNF-α between LGGs and HGGs. While NF-κB showed no statistically significant differences between various groups, the trending elevation in HGGs vs LGGs may reflect biological variability worth investigating in larger cohorts. The values of TNF-α, however, were significantly elevated in epileptic tissues compared to LGGs, consistent with previous studies linking seizure activity to heightened inflammatory responses. Mechanistically, the divergence in NF-κB and TNF-α expression patterns suggests a more complex regulatory network beyond the canonical adaptor protein myeloid differentiation factor 88 (MyD88)-dependent TLR pathway (48, 49). Moreover, TLR4 can also signal through a MyD88-independent, TRIF-dependent pathway, which may bypass NF-κB activation and explain the uncoupled expression patterns observed in our study (50). These results indicate the need to examine alternative TLR downstream mediators and cross-talk with other signaling pathways such as MAPKs or inflammasomes. In particular, future studies should assess apoptosis markers (e.g., Bax/Bcl-2) to explore TLR-mediated cell death mechanisms. Importantly, limitations such as small sample size and tissue heterogeneity may have impacted the results and should be addressed in future studies using larger cohorts and more refined, cell-type-specific analyses (51, 52). Exploring additional inflammatory mediators and apoptotic regulators could further clarify the contribution of TLR signaling in glioma and neuroinflammation.

**Table 1:** Patient data, including age, sex, and diagnosis, low-grade glioma (LGGs), high-grade glioma (HGGs), and epilepsy

Disease	Gender	Age (Years)	Location of the lesion
HGGs	Male	61	Left frontal lobe
HGGs	Male	5S8	Right parietal lobe
HGGs	Male	52	Left temporal lobe
HGGs	Female	72	Left frontoparietal lobe
HGGs	Female	50	Right parietal lobe
LGGs	Male	35	Right temporoparietal lobe
LGGs	Male	34	Left frontoparietal lobe
LGGs	Male	39	Left temporal lobe
LGGs	Male	21	Left frontal lobe
LGGs	Female	32	Left frontal lobe
Epilepsy	Female	19	Right temporal lobe
Epilepsy	Female	21	Left temporal lobe
Epilepsy	Female	38	Left temporal lobe
Epilepsy	Female	35	Right temporal lobe
Epilepsy	Male	21	Right Temporal lobe

Representative images taken from tissue passages P3 to P5. Scale = 100 μm

The data are displayed as the mean±SD, with statistical significance assessed at **P≤0.01

A) Representative Western blots showing the expression of NF-κB, TNF-α, TLR2, and TLR4 in tissue-derived primary cells from patients with LGG, HGG, and epilepsy. GAPDH was used as a loading control. B) Quantification of protein expression normalized to GAPDH. TLR2 expression was significantly higher in the HGG and epilepsy groups compared to LGG samples. TLR4 expression was significantly elevated in the epilepsy group compared to the LGG group. TNF-α level was significantly greater in the epilepsy group compared to LGG tissues. *P<0.05, *P<0.01; ns = not significant

## Conclusion

Our research underscores the varying levels of TLR2 and TLR4 expression in gliomas and epilepsy, with TLR2 showing a significant increase in HGGs and both TLRs exhibiting marked elevation in epilepsy tissues compared to LGGs. Although the levels of NF-κB and TNF-α did not consistently align with TLR expression across all groups, their partial increase suggests the need for further exploration. It is crucial to acknowledge that limitations such as small sample size and tumor heterogeneity may have impacted the results, which should be addressed in future research by utilizing larger patient populations and more precise analyses focused on specific cell types. Investigating additional inflammatory mediators and apoptotic regulators may provide further insights into the role of TLR signaling in glioma development and neuroinflammation. 

## Data Availability

Data are available upon request

## Declaration

We have not used any AI tools or technologies to prepare this manuscript.
